# Athletic performance, sports experience, and exercise addiction: an association study on *ANKK1* gene polymorphism rs1800497

**DOI:** 10.3389/fpsyg.2023.1182575

**Published:** 2023-07-31

**Authors:** Isık Bayraktar, Ladislav Cepicka, Magdalena Barasinska, Hasan Huseyin Kazan, Erdal Zorba, Mehmet Ali Ergun, Özgür Eken, Halil İbrahim Ceylan, Celal Bulgay, Tomasz Gabrys

**Affiliations:** ^1^Faculty of Sports Sciences, Alanya Alaaddin Keykubat University, Alanya, Türkiye; ^2^Department of Physical Education and Sport, Faculty of Education, University of West Bohemia, Pilsen, Czechia; ^3^Department of Health Sciences, Jan Dlugosz University, Czestochowa, Poland; ^4^Faculty of Medicine, Near East University, Mersin, Türkiye; ^5^Faculty of Sports Sciences, Gazi University, Ankara, Türkiye; ^6^Faculty of Medicine, Gazi University, Ankara, Türkiye; ^7^Department of Physical Education and Sport Teaching, Faculty of Sports Sciences, Inonu University, Malatya, Türkiye; ^8^Department of Physical Education of Sports Teaching, Faculty of Kazim Karabekir Education, Atatürk University, Erzurum, Türkiye; ^9^Faculty of Sports Sciences, Bingol University, Bingol, Türkiye

**Keywords:** *ANKK1*, athletics, athletic performance, exercise addiction, sport experience, rs1800497 polymorphism

## Abstract

**Introduction:**

Exercise addiction is a phenomenon being able to affecting the athletic performance. The gene, *ANKK1* and the polymorphism NM_178510.2:c.2137G > A (rs1800497) has been linked to the exercise addiction. However, further studies on diverse populations and sport branches are needed to totally explore the possible association of this polymorphism with the athletic performance. Thus, the present study aims to decipher any possible relations of the rs1800497 polymorphism with the athletic performance/personal best (PB) and sport experience of elite athletes.

**Methods:**

Sixty volunteer elite athletes (31 sprint/power and 29 endurance) and 20 control/sedentary participated in the study. The polymorphism was genotyped using whole exome sequencing approach and PB were determined according to the International Association of Athletics Federations (IAAF) score.

**Results:**

Our results underlined that there were not any significance differences for both allele and genotype frequencies between the groups in terms of athletic performance, although the frequency of allele G was higher (*p* > 0.05). Nevertheless, sport experience significantly associated with the rs1800496 polymorphism (*p* < 0.05).

**Discussion:**

In conclusion, genotype G/G could be inferred to be linked to the higher sport experience and athletic performance. Still, further studies with higher number of participants are needed to conclude the association of this polymorphism with athletic parameters.

## 1. Introduction

Since the popularity of sports increases on the whole world, the importance of athletic performance and the factors affecting the performance also advance (Bulgay et al., 2023). Athletic success has been shown to be linked to factors such as society, environment, psychology, and training patterns. Recently, individual characteristics including anaerobic and anaerobic metabolisms have been underlined to be linked to the genetic background. Thus, the current research on athletic performance has focused on the genetic variants significantly contributing to individual performance (Varillas-Delgado et al., 2022). In the late 1990s and early 2000s, research efforts were directed towards identifying specific single nucleotide polymorphisms (SNPs) associated with predisposition to certain sports and exercise-related phenotypes (Semenova et al., 2023). This field of study is commonly referred to as exercise or sports genomics. Several genes have extensively been studied in relation to athletic performance and exercise adaptation (Ahmetov and Fedotovskaya, 2015; Varillas-Delgado et al., 2022). The genes *ACE, ACTN3,* and *PPARGC1A* have been among the most commonly investigated ones in this context. Even though multifactorial approaches are required, it is critical to illuminate the associations of the genes and the variations on these genes with the athletic parameters (Cerit, 2018; Hall et al., 2023; Yang et al., 2023).

Recent studies have suggested that athletic performance or individual characteristics were affected by exercise addiction as well (Cetin et al., 2021; Szabo and Ábel, 2022). The definition of addiction has in the past been limited to the use of drugs and alcohol. Today, however, many behaviors such as exercise, sex, gambling, playing video games and using the internet are considered as potential addictive behaviors. Indeed, when beneficial behaviors become obsessive and excessive, it can lead to various negative outcomes (Szabo and Griffiths, 2007; De La Vega et al., 2016). Moreover, exercise addiction refers to a compulsive engagement in physical activity that is accompanied by a loss of control, withdrawal symptoms, and negative consequences, similar to other addictions (Cicioğlu et al., 2019; Liu et al., 2019). On the other hand, athletic performance refers to an individual’s ability to excel in a specific sport or exercise (Juwono et al., 2022). There can be a complex relationship between exercise addiction and athletic performance. While exercise addiction can lead to excessive training, which may improve athletic performance in short periods, the negative consequences of exercise addiction can ultimately lead to poorer athletic performance. Moreover, exercise addiction can result in injuries, burnout and fatigue, which can impair an individual’s ability to train and perform optimally (Godoy-Izquierdo et al., 2021). Additionally, exercise addiction can negatively impact an individual’s mental health, leading to decreased motivation and focus, which can also hinder athletic performance. Therefore, while exercise addiction and athletic performance can be related, it is essential to maintain a balance between training, and recovery and to seek professional help if addiction symptoms are present (Murakami et al., 2017; Michałowska-Sawczyn et al., 2021; Antrobus et al., 2022a,b).

Exercise addiction may stand out among the factors that have a negative impact on athlete performance. In the relevant literature, it has been observed that different results related to exercise addiction have been found. For example, in a systematic review, the athletes from endurance sports seem associated with the highest risk for exercise addiction (Di Lodovico et al., 2019). Nonetheless, exercise addiction can occur in any type of physical activity or sport. In a study conducted with a sample of Turkish track and field athletes, it was related that the athletes with high levels of exercise addiction displayed poor performance in all branches (Cetin et al., 2021). Another study indicated that loneliness and anxiety may lead to withdrawal and uncontrolled behavior that in turn leads to increased amount of exercise in amateur runners (Lukács et al., 2019).

The genes whose products are related to the dopaminergic system are linked to the addiction profile (Suchanecka et al., 2020). Neurotransmitter substances such as serotonin, and dopamine and the genes that metabolize them are fundamental in determining the effect of psychological factors on athletes. Those substances play a crucial role in the mental health, and addiction and thus physical activity (Shimura et al., 2023; Tanaka et al., 2023) by influencing the grey matter volume or white matter integrity in specific brain regions acting in the reward processing and decision making (Cui et al., 2022; Rajkumar, 2023). Hence, focusing on the genes involved in the dopaminergic system could be informative in the exercise addiction in the frame of the sport genetics.

The gene encoding Ankyrin repeat and kinase domain containing 1 (*ANKK1*) is amongst the genes linked to the addiction. *ANKK1* c.2137G > A (rs1800497) polymorphism was further shown to associate with the addiction specifically. The allele A has previously been associated to the altered cognitive behavioral capacity by changes in D2 receptor expression (Thompson et al., 1997; Lachowicz et al., 2020; Antrobus et al., 2022a,b). Moreover, the genotypes A/A and G/A have been linked to higher risk of certain neuropsychiatric disorders including schizophrenia and emotional eating habits, and obesity and alcohol, nicotine, and drug addiction (Aliasghari et al., 2021; Habibzadeh et al., 2021; Switala and Leonska-Duniec, 2021; Switala et al., 2022). Nevertheless, further studies are needed to comprehensively understand the relationship between this variant and the athletic parameters.

The aim of the present study is to compare the genotype and the allele frequencies of *ANKK1* rs1800497 polymorphism between elite sprinter/power and long-distance athletes in the presence of sedentary controls. The secondary aim of this study is to investigate possible associations between the rs1800497 polymorphism with the athletic performances and sports experience. To our knowledge, this is the first study investigating the association of rs1800497 polymorphism in elite track and field athletes. We hypothesized that significant differences in genotype and allele frequencies of the *ANKK1* rs1800497 polymorphism are expected between sprint/power and endurance athletes, and the G/G genotype is expected to have both better athletic performance and higher sports experience.

## 2. Materials and methods

### 2.1. Participants

The study was conducted in accordance with the Declaration of Helsinki and approved by the Gazi University Non-Interventional Clinical Research Ethics Committee (decision dated April 05, 2021, number 09). A total of sixty elite athletes (mean age = 25.07, SD = 4.80; years mean height = 174.97, SD = 7.89; cm mean body weight = 72.50, SD = 22.40; mean sports experience = 9.40, SD = 4.80; mean personal best = 1005.63, SD = 94.55) whom were affiliated with the Turkish Athletics Federation (TAF) and licensed in different clubs participated in this study. The inclusion criteria of the athletes were determined as that they had achieved a national ranking within the top ten in their respective sports disciplines and participated in International competitions such as the Olympic Games, European Championships, Universidad, Mediterranean Games, and Balkan Championship. The athletes were classified into two groups based on the characteristics of their events, namely sprint/power and endurance athletes. This classification was determined by considering parameters including distance, duration, and energy requirements of their branches. The sprint/power athlete group (*n* = 31) included athletes participating in events with primarily anaerobic energy demands such as 100–400-meter runners (*n* = 9), jumpers (*n* = 3), and throwers (*n* = 19). The endurance athlete group (*n* = 29) included athletes competing in long-distance events predominantly depending on aerobic energy production such as 3,000-meter (*n* = 12), 5,000-meter (*n* = 5), 10,000-meter (*n* = 4), and marathon (*n* = 8) runners. Additionally, a control group consisting of 20 individuals (mean age = 23.51, SD = 7.13 years) was included in the study. The inclusion criteria of the control group were settled as that they were without any known diseases in their families and any competitive sports experiences. The exclusion criteria were the withdrawal of the participants from the study and family connection between the participants.

#### 2.1.1. Sample collection

Before the measurements, participants were informed about the procedures and a voluntary consent form in the presence of a demographic information was obtained. For the molecular studies, 4 mL peripheral blood was obtained from each individual in the hospitals where the participants underwent routine check-ups during the preparation season period. The date of blood sample collection and corresponding code numbers were recorded. The blood samples were promptly transported in a cold chain to the Medical Genetics Laboratory at Gazi University, where the analyses were conducted.

### 2.2. Athletic performance

In order to assess the performance levels of the athletes in both groups, the International Association of Athletics Federations (IAAF; new name World Athletics) score scale was employed in both groups (sprint/power and endurance athletes), depending on their PBs. The IAAF scales are useful for the determination of performances of athletes from diverse athletics events and genders. For instance, the IAAF scale score of a male athlete who runs 100 m in 10.05 s is 1189, while that of a marathon runner who completes the race in 2 h 20 min 11 s is 997. Thus, the performance scale of the marathon runner is less than that of the 100 m runner (Spiriev, 2017).

### 2.3. Whole exome sequencing

The participants’ peripheral blood was processed using the DNeasy Blood and Tissue Kit (Qiagen, United States) to isolate total DNA, following the manufacturer’s instructions. The quality of the isolated DNA was assessed using a 1% agarose gel, and the concentration was measured using a NanoDrop (NanoDrop 1,000 Spectrophotometer V3.8; Thermo Scientific, United States). The Twist Human Comprehensive Exome Panel (Twist Biosciences, United States) was used for library preparation prior to WES, following the supplier’s instructions. Enzymatic DNA fragmentation was performed, and Twist Hybridization probes, as well as Dynabeads MyOne Streptavidin T1 (Invitrogen, United States), were used for hybridization. Following library enrichment and determination of library sizes, the samples were loaded onto flow cells and run on the Illumina NextSeq500 (Illumina Inc., United States). The minimum average read depth targeted was 200X. Raw data were processed using the HaplotypeCaller program from the Genome Analysis Toolkit (GATK) to obtain Binary Alignment Map (BAM) files, which were subsequently used to generate an output Variant Call Format (VCF) file using the GRCh38/hg38 reference genome. Finally, ANNOVAR (Wang, 2010; Van der Auwera et al., 2013) was used to annotate the variants.

### 2.4. Statistical analyses

Data analyses were conducted using Statistical Package for Social Sciences (SPSS) for Windows 25.0. Descriptive statistics, including numerical, percentage, mean, and standard deviation (SD) measures, were used to assess the data. The degree of heterogeneity between the results was evaluated using the Skewness and Kurtosis test (Kline, 2011). Genotype and allele frequencies for the polymorphism were calculated, and Hardy–Weinberg equilibrium (HWE) was assessed using either the chi-square (χ2) or Fisher’s exact test. The association between *ANKK1* rs1800497 polymorphism and sports experience was examined using one-way analysis of covariance (ANCOVA), with age as a covariate. Cohen’s d effect size (ES) with 95% confidence interval was calculated to define the magnitude of pairwise comparisons for pre-and post-test. The ES magnitude was defined as follows: <0.2 = trivial, 0.2 to 0.6 = small effect, >0.6 to 1.2 = moderate effect, >1.2 to 2.0 = large effect, and > 2.0 = very large (Hopkins et al., 2009). Data were significant when *p* < 0.05.

## 3. Results

**Figure 1:**
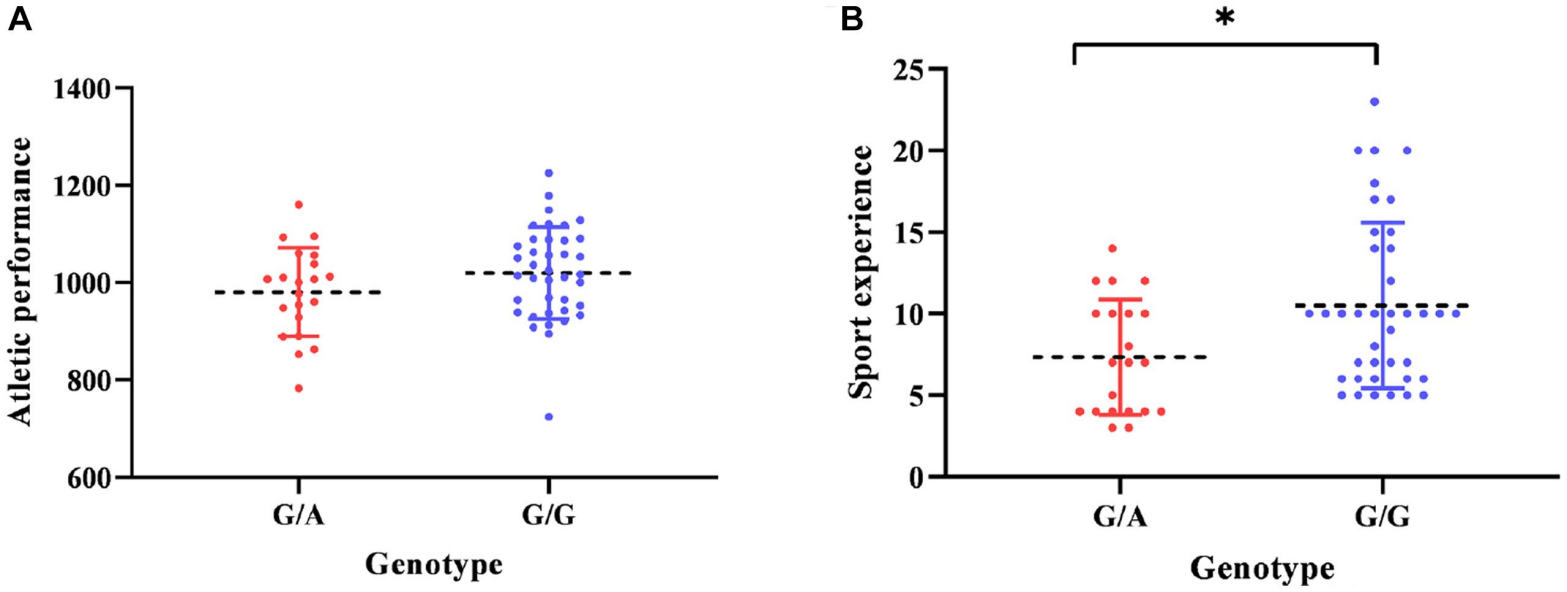
The relationship between **(A)** athletic performance, **(B)** sport experiences and two different genotypes (G/A and G/G), *p < 0.05.

The present study aims to assess any possible associations between rs1800497 polymorphism and the athletic parameters in Turkish elite sprint/power and endurance athletes in the presence of control group. According to the results, the genotype frequencies of rs1800497 polymorphism was in concordance with the Hardy–Weinberg equilibrium in both athletes and control individuals. Although the number of the allele G was higher compared to the allele A within each group, there were not any significant differences in genotype (*p* = 0.461) and allele distribution (*p* = 0.540) between the groups (Table 1). Additionally, no significant differences for the association of the rs1800497 polymorphism with competitive performance were detected [*t* = 1.547, *p* = 0.127, standardized effect size: 0.42, moderate effect (Table 2; Figure 1A)]. Critically, the association of the rs1800496 polymorphism with sports experience was statistically significant [*t* = 2.555, *p* = 0.013, standardized effect size: 0.72, moderate effect (Table 3; Figure 1B)].

**Table 1:** Genotype and allele frequencies of *ANKK1* rs1800497 polymorphism in Turkish elite athletes and controls.

	Genotype			*p*-Value	Allele		*p*-Value
	G/G	G/A	A/A	0.461	G	A	0.540
Sprint/Power	18 (58.1%)	13 (41.9%)	–		49 (79.0%)	13 (21.0%)	
Endurance	21 (72.4%)	8 (27.6%)	–		50 (86.2%)	8 (13.8%)	
Controls	14 (70.0%)	6 (30.0%)	–		34 (85.0%)	6 (15.0%)	

**Table 2:** Association of the *ANKK1* rs1800497 polymorphism with the athletic performances.

Genotype	*n*	*M*	SD	*t*	*p*	Cohen’s d
G/G	39	1019.33	94.46	1.547	0.127	0.42
G/A	21	980.19	91.49			
A/A	–	–	–			

**Table 3:** Association of the *ANKK1* rs1800497 polymorphism with the sport experiences.

Genotype	*n*	*M*	SD	*t*	*p*	Cohen’s d
G/G	39	10.51	5.07	2.555	0.013*	0.72
G/A	21	7.33	3.52			
A/A	–	–	–			

N: Number; M: Mean; SD: Std. Deviation; *Statistically significant differences (*p*<0.05); adjusted by age.

## 4. Discussion

In the present study, we investigated the genotype and allele frequencies of the *ANKK1* rs1800497 polymorphism between the elite sprint/power, endurance athletes and matched controls and possible association of this polymorphism with the athletic performance, and sports experience. To our best knowledge, the current study is the first investigation to determine whether the rs1800497 polymorphism influences athletic performance and sports experience of elite athletes in Turkish population in a perspective of the exercise addiction-associated gene, *ANKK1*.

According to our results, that there were not any statistically significant differences in allele and genotype frequencies of the *ANKK1* rs1800497 polymorphism between sprint/power athletes, endurance athletes, and the control group. Despite insignificance, wild-type (G/G) genotype distribution was higher compared to other ones within each group (Table 1). Michałowska-Sawczyn et al. (2021) also obtained similar findings, showing that the number of athletes with the G/G genotype was higher, although there were no significant differences observed in their study that included 258 sportsmen and 284 control individuals. Similarly, in another study involving elite rugby players, it was reported that the prevalence of the G/G genotype was higher (Antrobus et al., 2022a,b). The hypothesis of the present study was not accepted; nonetheless, it can be said that the obtained findings are similar to the literature. It could be due to several reasons which would be low sample size, insufficient methodological issues, conflicting evidences, unforeseen factors and incomplete understanding of the multi-genetic background. Our research group is currently conducting ongoing research to address and resolve these issues for future studies.

In terms of the athletic performance, it was observed that athletes’ performances did not reveal any significant correlations with the polymorphism although it was found that the athletes with the G/G genotype tended to have higher PBs than those with the G/A genotype (Table 2). In recent years, genetic factors have begun to emerge as key research topics in addition to environmental factors in terms of influencing the athletic performance. Studies in the field of sport genetics have indicated that while individuals could reach a certain level of optimal performance through accurate and systematic application of training, the level of performance development may be limited by their genetic capacity (Yıldırım et al., 2022). Moreover, specific polymorphisms in the genes encoding such as Alpha-actinin-3 (*ACTN3*), Angiotensin I-converting enzyme (*ACE*), Adenosine monophosphate deaminase-1 (*AMPD1*), Bradykinin receptor B2 (*BDKRB2*), Endothelial nitric oxide synthase 3 (*NOS3*), Alpha-2A adrenergic receptors (*ADRA2A, ADRA2B* and *ADRA2C*), Peroxisome proliferator-activated receptors (*PPAR*), Peroxisome proliferator-activated receptor gamma coactivator 1-alpha (*PPARGC1A*), Vitamin D receptor (*VDR*), Erythropoietin (*EPO*), and C-reactive protein (*CRP*) have been emphasized to be associated with athlete specialization and performance. The involvement of the proteins encoded by the mentioned genes in the muscular system, cellular and systemic metabolism and oxygenation makes any variations that may occur on these genes and subsequent changes in the proteins valuable even if it is not directly associated with diseases (Varillas-Delgado et al., 2022). In addition to the listed genes, *ANKK1* rs1800497 polymorphism influences cognitive behavioral capacity by the modulation of expression of D2 receptors (Thompson et al., 1997). Thus, this polymorphism has been included in the group of key variants involved in the process of addiction (Lachowicz et al., 2020). Moreover, some studies have emphasized that exercise addiction was associated with athletic performance. For instance, Cetin et al. have showed that the athletes with high exercise addiction gave the lower performances independent of the branches (Cetin et al., 2021). The further studies by Cicioğlu et al. (2019) underlined that elite athletes had a higher risk of being exercise addicted group pointing that there would be a correlation between exercise addiction and enhanced athletic performance. In terms of training, a study reported the association of *ANKK1* rs1800497 polymorphism with the exercise habit in the period from childhood to adolescence in Japanese population. The study further figured out that the individuals with A/A genotype were with lower levels of dopamine receptor, resulting in affected exercise habits (Murakami et al., 2017). Recent studies have reported that individuals with the A/A or A/G genotype may be at higher risk for addictive behaviors, including exercise addiction, while those with the G/G genotype may have a lower risk for addictive behaviors (Aliasghari et al., 2021; Habibzadeh et al., 2021; Switala and Leonska-Duniec, 2021; Switala et al., 2022).

In current study, there were not any individuals with A/A genotype. Hence, it was difficult to evaluate the exercise addiction and comment on the performances in the elite athletes. However, the lack of A/A genotypes and high number of G/G genotype may indicate that the individuals in our study could not tend to addict on the exercise/training. Still, more studies with exercise addiction scale and higher number of participants should be conducted to fully understand the role of A/A genotype on exercise addiction and correspondingly athletic performances in Turkish population. Furthermore, it is important to acknowledge that the relationship between *ANKK1* genotype and addiction is likely a complex phenomenon involving multigenetic and environmental factors, and it is not solely determined by a single gene. Overall, our hypothesis pointing the possible association between the rs1800497 polymorphism and athletic performance fails and multi-genetic approaches are needed because of the complexity of the performance-determining mechanisms.

Importantly, the present study indicated that sport experiences significantly associated with the rs1800497 polymorphism. The relevant literature review indicates that sport experience or sport status has been identified as a significant factor in elite rugby (Heffernan et al., 2016), and basketball players (Demirci et al., 2023). Sport experience can significantly vary across different sports disciplines. Indeed, the performance development of marathon runners can be influenced by a combination of environmental and genetic factors, as well as sports experience. However, in sprint disciplines, athletes may retire from sports at earlier ages, and a notable example of this is Usain Bolt, the Olympic record holder in the 100 m event. Conversely, endurance is a bio-motor characteristic that develops over time and different from sprinters where muscular strength plays a crucial role. By aging, there can be a decline in neuromuscular junctions and a decrease in muscle strength, which can negatively impact sprinters’ performance (Van Gent et al., 2007; Michaelis et al., 2008; Gómez et al., 2013). Due to the limited number of studies investigating the association between *ANKK1* gene polymorphism and performance, and sports experience, it is challenging to make definitive conclusions in this regard. However, it is believed that in addition to field and laboratory performance tests specific to each sport, the inclusion of genetic analyses in evaluating athletes’ performance and training practices could contribute to their high-performance levels more efficiently.

## 5. Conclusion

The results of the study indicated that there was no significant association between *ANKK1* gene rs1800497 polymorphism and athletic performance. However, when the sports experience was considered, significant association was found, suggesting that the rs1800497 polymorphism may serve as a marker for predicting the length of an elite athlete’s professional career. In future research, increasing the sample size and investigating the relationship between this polymorphism and various performance characteristics (such as aerobic capacity, strength, muscular endurance, and body composition) in both male and female athletes competing in different sports could be explored in greater details. Still, according to the current study, it is suggested that the genetic background can be utilized to guide athletes during the professional management period, particularly in the presence of physical factors. This means that understanding an athlete’s genetic profile can provide valuable information for personalized training programs, injury prevention strategies and optimizing performances based on individual genetic predispositions. By incorporating genetic information into the management and guidance of athletes, it may be possible to enhance their overall development and maximize their potential in a more targeted and tailored manner.

## 6. Limitations and future directions

The current study has several limitations that should be acknowledged. Firstly, the sample size of the study is small, which makes it challenging to generalize the findings. Secondly, a structured scale or questionnaire was not used to measure exercise addiction. In future studies, the inclusion of various scales and questionnaires could provide further support for the obtained results. Thirdly, only one polymorphism was utilized in the present research which restricts the cumulative evaluation of the genetic background. Despite these limitations, the current study highlighted the genotype and allele frequencies of the *ANKK1* rs1800497 polymorphism among sprint/power and endurance athletes and demonstrated a potential relationship between the polymorphism and sports experience.

## Data availability statement

The datasets presented in this study can be found in online repositories. The names of the repository/repositories and accession number(s) can be found at: https://figshare.com/, https://doi.org/10.6084/m9.figshare.23254490.v2.

## Ethics statement

The studies involving human participants were reviewed and approved by Gazi University Non-Interventional Clinical Research Ethics Committee (decision dated April 05, 2021, number 09). The patients/participants provided their written informed consent to participate in this study.

## Author contributions

IB and CB: conceptualization. HK and ME: methodology. EZ and MB: software. ME, TG, and LC: validation. CB and IB: formal analysis. CB, ÖE, and HC: investigation. LC, MB, and TG: resources. IB and HK: data curation. CB, LC, MB, and IB: writing—original draft preparation. CB, ME, HK, HC, and TG: writing—review and editing. CB and HK: visualization. ME: supervision. EZ: project administration. All authors contributed to the article and approved the submitted version.

## Funding

The study was supported by Gazi University Rectorate (Scientific Research Projects Coordination’s Unit, Project number: TCD-2021-7116). Published with the financial support of the European Union, as part of the project entitled Development of capacities and environment for boosting the international, intersectoral, and interdisciplinary cooperation at UWB, project reg. no. CZ.02.2.69/0.0/0.0/18_054/0014627.

## Conflict of interest

The authors declare that the research was conducted in the absence of any commercial or financial relationships that could be construed as a potential conflict of interest.

## Publisher’s note

All claims expressed in this article are solely those of the authors and do not necessarily represent those of their affiliated organizations, or those of the publisher, the editors and the reviewers. Any product that may be evaluated in this article, or claim that may be made by its manufacturer, is not guaranteed or endorsed by the publisher.
